# Case report and literature review: cardiac hematic cyst

**DOI:** 10.3389/fcvm.2024.1417074

**Published:** 2024-07-30

**Authors:** Roberto Baltodano-Arellano, Eduardo Alvarez-Tiburcio, Lucia Barriales-Revilla, David Bellido-Yarlequé, Angela Cachicatari, Kelly Cupe-Chacalcaje, Alan La Torre-Zuñiga, Kevin Velarde-Acosta

**Affiliations:** ^1^ Cardiac Imaging Area of Cardiology Service, Hospital Guillermo Almenara Irigoyen – EsSalud, Lima, Peru; ^2^ School of Medicine, Universidad Nacional Mayor de San Marcos, Lima, Peru; ^3^ Cardiac Surgery Unit, Hospital Guillermo Almenara Irigoyen - EsSalud, Lima, Peru; ^4^ Clinical Cardiology Service, Hospital Guillermo Almenara Irigoyen - EsSalud, Lima, Peru; ^5^ Pathological Anatomy Service, Hospital Edgardo Rebagliati Martins - EsSalud, Lima, Peru

**Keywords:** right atrium, cardiac hematic cyst, echocardiography, computed tomography angiography, multimodality imaging

## Abstract

A 49-year-old female patient, asymptomatic, presented to the cardiology office for a right atrial mass, identified incidentally in a non-electrocardiogram (ECG)-gated contrast-enhanced computed tomography, performed for follow-up of pulmonary tuberculosis. Echocardiography, surprisingly, showed an anechogenic ovoid mass in the right atrium measuring 40 × 40 mm^2^, implanted in the interatrial septum without affecting the tricuspid valve. ECG-gated computed tomography angiography (CTA), confirmed the dimensions of the mass, which presented homogeneous content, calcified areas, and a 12-mm pedicle implanted near the ostium of the coronary sinus. Additionally, contrast uptake and infiltration of adjacent structures were ruled out. In the surgical field, an encapsulated mass with blood content was found, which pathology reported as a hematic endocardial cyst (HEC). These are rare cardiac masses, constituting 1.5% of all primary cardiac tumors. It is usually an incidental finding, and its clinical presentation will depend on its dimensions and the intracardiac hemodynamic impact. A highlighting feature is its anechogenic content on ultrasound, however, multimodality imaging allows for making diagnostic assumptions, discerning between primary cardiac tumors, and provides morphological and hemodynamic information useful for therapeutic decision making. The age of the patient, the large size of the HEC, and its location in the interatrial septum make up a completely atypical presentation of this rare disease, which motivated this report.

## Case report

### Clinical presentation

A 49-year-old woman from the Peruvian Andes presented to the cardiology office due to an incidental tomographic finding of a mass in the right atrium. In the anamnesis, the patient reported being asymptomatic, while the cardiorespiratory physical examination did not show relevant findings. Her medical history was notable for a tuberculous pulmonary nodule removed 2 years previously, for which she received complete treatment for 6 months and required subsequent computed tomography (CT) controls. She had no cardiovascular risk factors or relevant family or socioeconomic history. Laboratory tests were within normal ranges, while the electrocardiogram showed no pathological alterations.

### Differential diagnosis

In the presence of a right atrial mass implanted in the interatrial septum, a myxoma should be considered due to its frequency (1–3). Echocardiography and magnetic resonance imaging (MRI) determine structural and tissue characteristics that bring the diagnosis closer. On some occasions, the finding of a thrombus trapped in the foramen ovale has been described in transesophageal echocardiography (TEE) (4, 5). This mass's cystic appearance guides the diagnosis of rarer pathologies such as cardiac hydatid, bronchogenic or endocardial hematic cyst, which are usually diagnosed in pathological anatomy (6–8).

### Diagnostic workup

**Figure 1:**
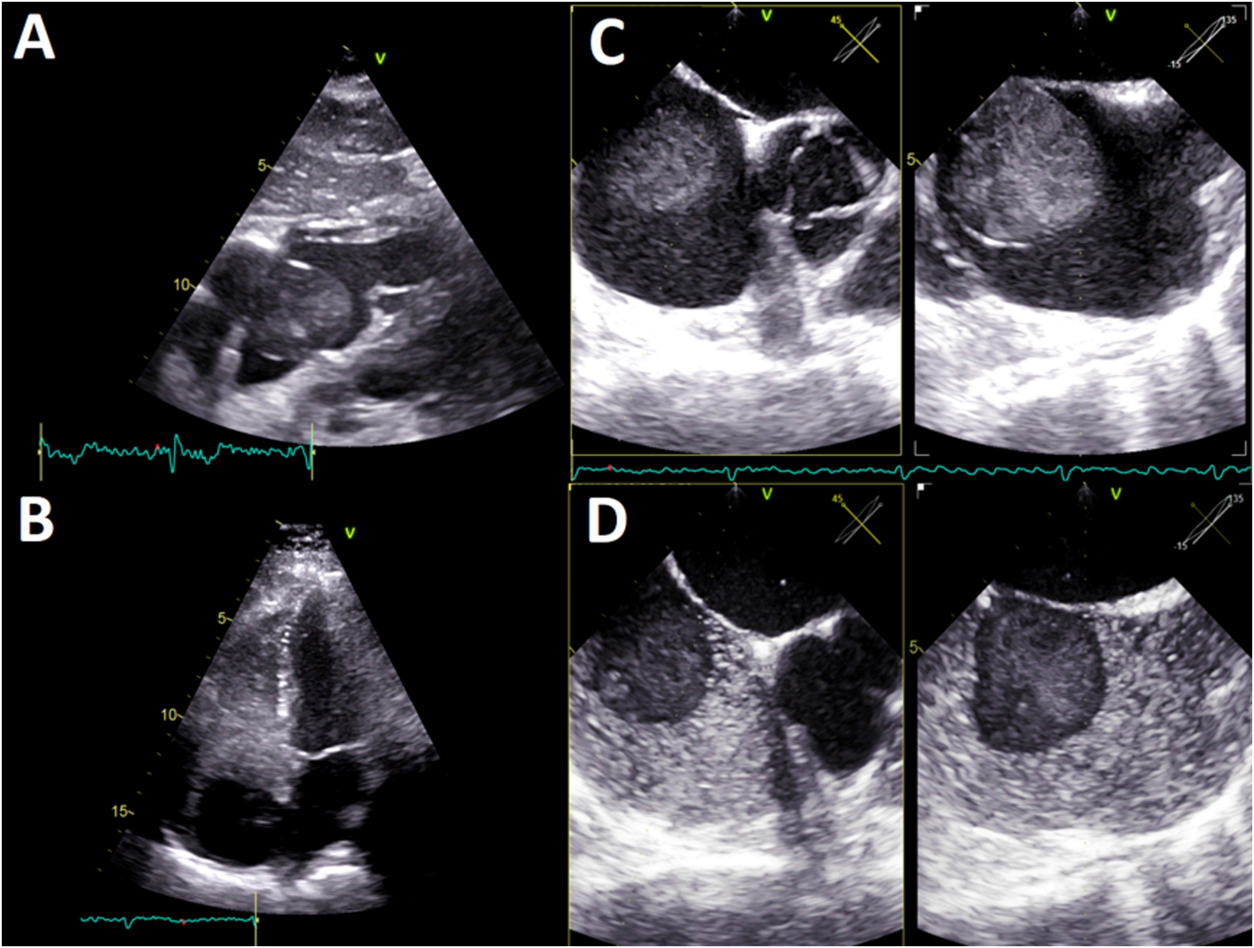
(**A**) TTE, four-chamber subcostal view. Ovoid mass in the right atrium, with echogenicity almost blood-like and calcification areas. (**B**) TTE, four-chamber apical view. The agitated saline test shows an anechogenic, 40 × 40 mm^2^, mass in the right atrium implanted in the interatrial septum. No tricuspid valve compromise was observed. (**C**) TEE, multiplanar image of the interatrial septum (45°; 135°). A homogeneous pedunculated mass with a thin covering was implanted in the middle-low interatrial septum with pendulum movement. (**D**) TEE, multiplanar image of the interatrial septum (45°; 135°). The agitated saline test depicts anechogenicity of the hematic mass. TTE, transthoracic echocardiography; TEE, transesophageal echocardiography.

A cardiologic study plan was initiated with transthoracic echocardiography (TTE), which confirmed the presence of an anechogenic ovoid mass in the right atrium measuring 40 mm × 40 mm^2^, implanted in the interatrial septum without affecting the tricuspid valve (Figure 1A; Supplementary Video S1). In the study with agitated saline solution, echogenicity of the blood-like mass was evident (Figures 1B,D, Supplementary Video S2). No additional relevant findings were found.

**Figure 2:**
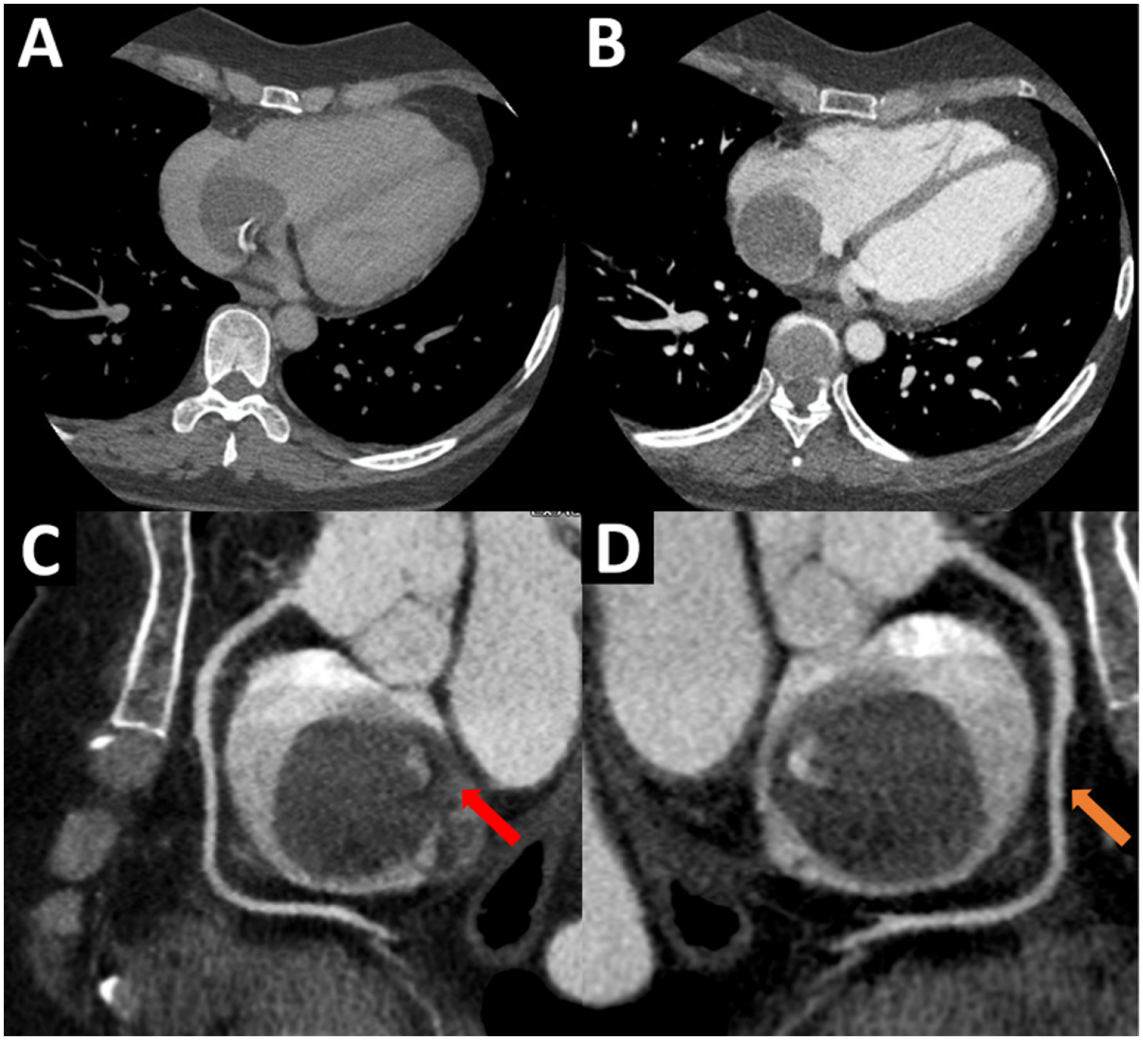
(**A**,**B**) ECG-gated CTA, four-chamber view. A mobile, round, homogenous mass is seen adhered to the right lower interatrial septum. (**C**,**D**) ECG-gated CTA, sagittal views of the right atrium. A homogeneous, non-contrast-enhancing mass with calcified areas and defined edges is seen in the right atrium. Note the 12 mm pedicle (red arrow) that attaches it to the interatrial septum, near the fossa ovalis. Likewise, the right coronary artery (orange arrow) is seen, which does not provide collateral to the mass. ECG, electrocardiogram; CTA, computed tomography angiography.

The TEE showed a homogeneous mass covered by a thin layer, implanted in the mid-low septum, and exhibited pendulum movement (Figure 1C, Supplementary Video S3). Furthermore, the previous dimensions were confirmed and no signs suggestive of vascularization were found. Given these findings, the diagnosis work-up was complemented with an ECG-gated CTA, displaying an ovoid, mobile mass of 35 mm in diameter with homogeneous content and calcified areas with a 12-mm pedicle adhered to the lower atrial septum near the coronary sinus ostium (Figure 2).

### Treatment

**Figure 3:**
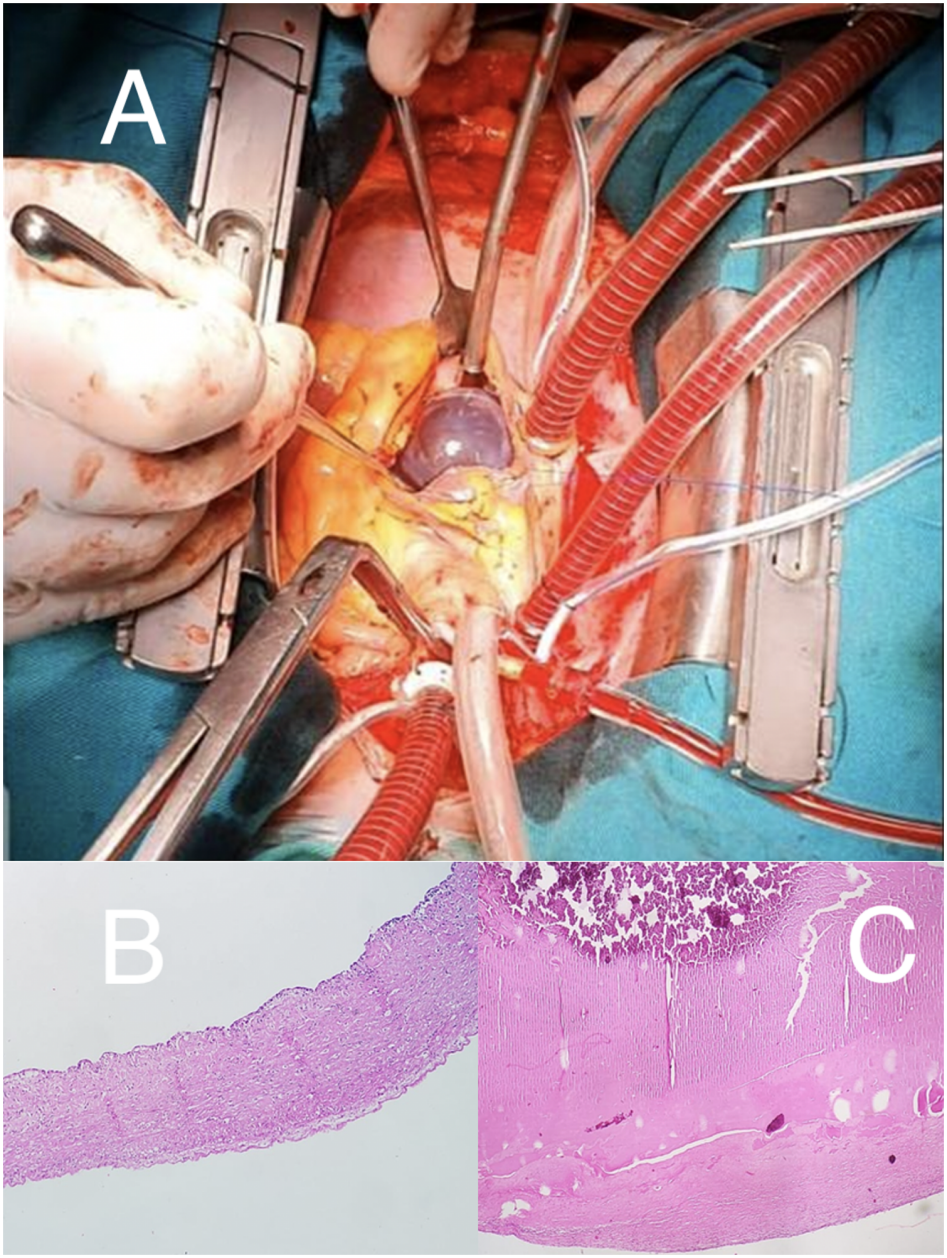
(**A**) Surgical field. Right atriotomy, with exposure of violaceous, smooth, and shiny mass. (**B**) Microscopy: fibrous (composed of collagenous layers) cyst wall without epithelial cells. Presence of some lymphocytes. (**C**) Microscopy: uniform fibrin content with calcification areas (upper region with more intense staining).

With these findings, the patient underwent an open surgical resection of the mass. After the right atriotomy, a violaceous, smooth, tense, and shiny mass was found, suggesting a cyst with bloody content (Figure 3A). The pathological study described a fibrous (collagenous) wall devoid of epithelium and with few inflammatory cells. Furthermore, fibrin content with areas of calcification was reported (Figures 3B,C), confirming the diagnosis of HEC (8–10).

### Follow-up

At one year of follow-up, the patient did not present relevant symptoms, except for a nonspecific chest pain in the healed surgical wound. Due to a suboptimal acoustic window, TEE was performed, which excluded mass recurrence.

## Discussion

HEC constitutes 1.5% of all cardiac tumors (8). It occurs mainly in infants and its preferential location is the heart valves (8, 9, 11). Its origin is still unknown, but two hypotheses are suggested: the first describes ectatic vessels evolving into a hematic cyst, and the second describes local inflammation that develops into a hematoma and transforms into a hematic cyst (8, 9).

To learn about clinical and therapeutic features in adults affected by this rare entity, we did an extensive search of the medical literature in Medline for articles published up to January 2022 (Table 1). Our search revealed that this disease occurs indistinctly in both sexes and the main location of this mass is the mitral valve, as it occurs in infants. Likewise, it allowed us to know that, in our case, the magnitude of the mass, located in an uncommon site, is the largest published in the literature.

**Table 1:** Reported cases of hematic cyst in adult patients.

Patient	Year of publication	Age	Gender	Size	Anatomical location	Sign and symptoms	Time course	Complications	Treatment	Reference
1	1983	27-year old	Male	25 mm	Mitral valve anterior leaflet Anterolateral papillary muscle	Acute right-sided Hemiparesis Expressive Aphasia	NR	None	Surgical excision Median sternotomy	(12)
2	1990	46- year old	Male	30 × 25 mm	Mitral valve anterior Leaflet	Chest tightness on exertion	5 years	None	Surgical excision Median sternotomy	(13)
3	1992	41-year-old	Female	13 × 10 mm 2 × 3 mm	Mitral valve posterior leaflet	Dyspnea on exertion	NR	None	Surgical excision of BC (13 × 10 mm) Median sternotomy	(14)
4	1995	16-year-old	Female	13 mm	Right aortic valve leaflet Free margin	Systolic ejection murmur left sternal border	2 years	None	Surgical excision Median sternotomy	(10)
5	1996	59-year-old	Female	20 × 20 mm	Interatrial septum	Substernal Pressure Systolic Murmur	NR	None	Surgical excision Median sternotomy	(15)
6	1999	50-year-old	Male	21 × 22 mm	Mitral valve anterior leaflet	Left Parasternal Systolic Murmur	NR	None	Conservative	(16)
7	2000	45-year-old	Female	20 × 20 mm	Mitral valve anterior leaflet	Asymptomatic	NR	None	Surgical excision Median sternotomy	(17)
8	2003	52-year-old	Male	40 × 30 mm	Interatrial septum	Asymptomatic	NR	None	Surgical excision Median sternotomy	(18)
9	2004	44-year-old	Female	20 mm	Mitral valve anterior leaflet	Dyspnea on exertion	NR	None	Surgical excision Median sternotomy	(19)
10	2005	25-year-old	Female	23 × 25 mm	Mitral valve anterior leaflet	Asymptomatic	NR	None	Surgical excision Median sternotomy	(20)
11	2005	35-year-old	Male	15 × 21 mm	Mitral valve anterior leaflet	NR	NR	None	Surgical excision	(21)
12	2006	65-year-old	Female	44 × 20 mm	Interatrial septum	Headache	NR	None	Surgical excision	(22)
13	2007	29-year-old	Male	30 mm	Mitral valve anterior leaflet	Chest pain	9 months	None	Surgical excision Median sternotomy	(23)
14	2008	62-year-old	Male	30 mm	Interatrial septum	Syncope Headache	NR	None	Surgical excision Median sternotomy	(11)
15	2008	62-year-old	Female	NR	Mitral valve anterior leaflet	Dyspnea on exertion Systolic Murmur	7 days	None	Surgical excision Median sternotomy	(24)
16	2009	65-year old	Female	10 × 10 mm 10 × 10 mm	Mitral valve anterior leaflet Mitral valve posterior leaflet	Chest pain	4 months	None	Surgical excision Median sternotomy Mitral valvuloplasty	(25)
17	2011	28-year-old	Female	19 mm	Mitral valve posterior leaflet	NR	NR	None	Surgical excision Median sternotomy	(26)
18	2011	69-year-old	Male	40 × 25 mm	Interatrial septum	Asymptomatic	NR	None	Surgical excision Median sternotomy	(8)
19	2012	55-year-old	Male	20 × 18 mm	Mitral valve sub valvular apparatus Posterior papillary muscle	NR	NR	None	Surgical excision Myocardial revascularization Median sternotomy	(27)
20	2012	47-year-old	Male	16 × 14 mm	Mitral valve anterior leaflet Anterolateral papillary muscle	Asymptomatic	3 weeks	None	Surgical excision Mitral valvuloplasty Median sternotomy	(28)
21	2013	25-year old	Male	NR	Mitral valve anterior leaflet	Dyspnea on exertion	NR	NR	NR	(29)
22	2015	23-year-old	Male	20 mm	Mitral valve anterior leaflet	Shortness of breath	NR	None	Surgical excision Trans-septal approach Mitral valvuloplasty Ring Annuloplasty	(30)
23	2015	70-year-old	Female	16 mm	Mitral valve anterior leaflet	Dyspnea on exertion	NR	None	Surgical excision Median sternotomy	(31)
24	2016	85-year-old	Female	30 × 30 mm 25 × 25 mm	Interatrial septum	Asymptomatic	NR	None	Surgical excision Median sternotomy	(32)
25	2019	47-year-old	Female	10 mm	Mitral valve sub valvular apparatus	Chest pain	3 days	None	Conservative	(33)
26	2020	57- year old	Female	10 × 10 mm	Mitral valve anterior leaflet (A1–A2 segment)	Chest pain Fever	NR	None	None	(34)

The set of symptoms depends on the mass dimensions and its impact on intracardiac hemodynamics. As cysts may involve the free edges of the valves, patients can present with dyspnea or heart murmurs due to valve regurgitation. Other clinical manifestations described are systemic embolism, syncope, or even sudden death (8, 9). Despite the multiple possibilities of clinical manifestations, the vast majority of findings of this tumor are incidental, as seen in our patient in whom the tumor was discovered in a tomographic control for pulmonary tuberculosis.

Within cardiac imaging studies, echocardiography is essential for the initial examination of intracardiac masses (1–3, 35, 36). It provides morphological information, data on the anatomical relationship, and determines the hemodynamic impact of the HEC. The cysts have particular ultrasound characteristics such as a thin reflective layer and an echolucent content, which could go unnoticed by novice explorers (9, 11). A meticulous analysis of our patient images confirmed these findings. The agitated saline solution study was useful because it highlighted the magnitude and dynamics of the mass, in addition to ruling out interatrial shunts. TEE describes with high precision anatomical aspects of atrial masses and defines carefully the components of a cyst, including the absence of vascularization, as seen in the images of this case (8, 9).

ECG-gated CTA reported a homogeneous mass with calcified areas and defined edges, mobile, without contrast-enhancing or infiltration. Also, it excluded the presence of additional intra-cardiac masses, contributing to better surgical planning (37). Cardiac MRI is an important technique to define contrast uptake in masses. In particular, hematic cysts do not capture medium contrast, because they are not vascularized, unlike a malignant neoplasm (9). Due to the cystic structure, hydatid disease must be ruled out through specific MRI sequences that differentiate it from the hematic cyst. In the former the T1 signal is hypointense and T2 signal hyperintense, while in the latter the T1 and T2 signals are isointense (11). Despite the usefulness of cardiac MRI for the differential diagnosis of cardiac masses, it was not performed in our patient because the magnetic resonator was inoperative during that period; likewise, surgical resection of the cyst had already been decided by the Heart Team based on ultrasound and tomographic features.

The decision to surgically remove an asymptomatic cardiac mass is based on avoiding embolic phenomena and ruling out malignancy. If the nature of this tumor is specified with imaging tests, the surgical time will depend on the speed of growth, hemodynamic impact, and the risks of rupture and embolization (8, 9, 38, 39). Although our patient did not present cardiac symptoms, the intervention was based on age, the low risk of malignancy, the dimensions of the mass and the prevention of embolisms, as occurred in the vast majority of cases reported in the literature.

## Conclusions

The HEC is an extremely rare mass that usually affects the heart valves of infants. The characteristics of this report, such as the adult age of the patient, the anatomical location in the interatrial septum, and the gigantic dimensions of the mass, are unprecedented in the medical literature.

Multimodality imaging allows differential diagnosis between primary cardiac tumors and provides useful morphological and hemodynamic information for therapeutic decision making. Surgical removal avoids embolic phenomena and hemodynamic disturbances. Finally, the purplish, smooth, shiny, blood-bag-like surgical piece is a distinguishable feature of this cardiac mass.

## Data Availability

The original contributions presented in the study are included in the article/Supplementary Material, further inquiries can be directed to the corresponding author.

## Supplementary material

Supplementary Video S1 TTE, subcostal view. Evidence of ovoid mass in the right atrium with mainly hypoechoic contents.

Supplementary Video S2 TTE, apical view 4 chambers. Agitated saline demonstrates a mass in the right atrium with echogenicity similar to blood.

Supplementary Video S3 TEE, X-plane. Saline solution test highlights pear morphology of mass implanted in the interatrial septum.

Supplementary Data Sheet 1 Timeline of patient evolution.
