# Supplementary figures and images for: Case report and literature review: cardiac hematic cyst
Source: Front Cardiovasc Med. 2024 Jul 30;11:1417074. doi: 10.3389/fcvm.2024.1417074 (PMC11319172; doi:10.3389/fcvm.2024.1417074)

## Timeline of patient evolution

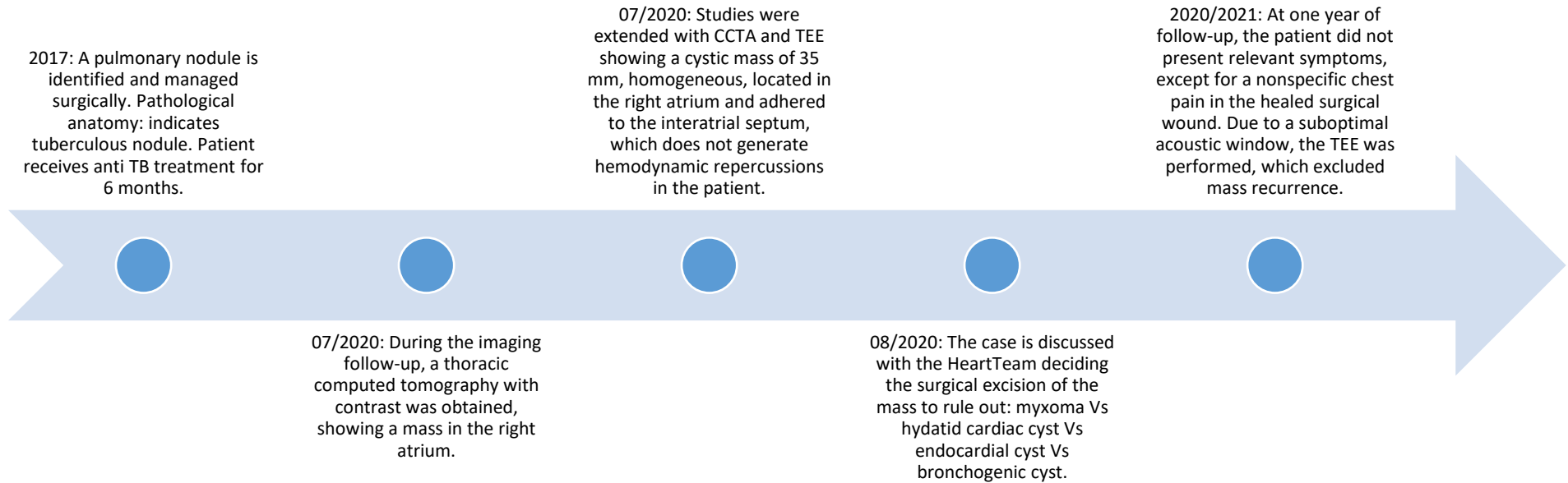

Supplement: Supplementary Data Sheet 1 — Timeline of patient evolution. [file Datasheet1.pdf]
